# A case of uveitis and retinal vasculitis induced by varicella-zoster virus: vitrectomy treatment and literature review

**DOI:** 10.1186/s12886-026-04710-2

**Published:** 2026-03-09

**Authors:** Jingjing Tian, Yuze Jiang, Nannan Ye, Yankun Zhang

**Affiliations:** 1 Hebei Chest Hospital, No. 372 Shengli North Street, Chang’an District, Shijiazhuang City, Hebei Province China; 2 Hebei Medical University, No. 361 Zhongshan East Road, Chang’an District, Shijiazhuang City, Hebei Province China

**Keywords:** Metagenomic next-generation sequencing, Varicella-zoster virus, Uveitis, Retinal vasculitis, Vitrectomy

## Abstract

**Background:**

Infectious uveitis can be induced by a variety of factors, including viral, bacterial, and parasitic infections, among others. Among these, viral infections are the most common cause of infectious uveitis. Traditional diagnostic methods have limited sensitivity and are often cumbersome, which restricts their ability to accurately identify the viral pathogens responsible. As a result, there is still no gold-standard diagnostic tool for detecting uveitis. With advancements in molecular diagnostic technologies, metagenomic next-generation sequencing (mNGS) has become widely used in clinical sample detection, molecular sequencing, and microbial analysis.

**Case report:**

This article reports a case of an elderly male patient who presented with a one-month history of left eye vision deterioration. The patient had a generally healthy status, with a 17-year history of hypertension, and denied any other underlying conditions. Ophthalmic examination showed planktonic cells (+++) in the anterior chamber of the left eye, partial posterior adhesion of the iris, lens opacity, visible attachment of iris pigments in front, severe vitreous opacity, visible flocculent yellow white floating material, and faintly visible optic disc structure in the fundus. These findings led to a diagnosis of left eye uveitis. Given the severity of the ocular condition, a decision was made to perform vitreous cavity puncture for smear culture and metagenomic mNGS to identify the pathogen. mNGS promptly detected 9939 sequence counts of Varicella-zoster virus (VZV), confirming the pathogen. Following the identification, Acyclovir injection is used for systemic intravenous injection and local intravitreal injection of ganciclovir injection for antiviral therapy.After two weeks of treatment, the symptoms of anterior uveitis showed some improvement, but the vitreous inflammation remained largely unchanged. Consequently, a vitreous body removal (vitrectomy) was performed, postoperative fundus examination showed the refractive interstitium is clear, and Kyrieleis plaques along the inner wall of the paraoptic artery can be seen. Postoperatively, the patient’s vision improved rapidly. Previous reports typically emphasize the importance of antiviral therapy. However, in this case, the authors discuss the use of vitrectomy in a VZV infection-induced pan-uveitis and retinal vasculitis patient, where antiviral therapy alone was insufficient, resulting in a satisfactory outcome post-surgery.

**Conclusion:**

Intraocular fluid metagenomic testing is an effective method for diagnosing unexplained uveitis. For patients with VZV-associated uveitis and severe vitreous opacity, early vitrectomy is an effective therapeutic approach.

Varicella-Zoster Virus (VZV) is a double-stranded DNA virus belonging to the alpha-herpesvirus family. It exhibits neurotropism and the ability to undergo latent reactivation [1]. Primary infection with VZV typically leads to chickenpox [2]. After the initial infection, VZV remains latent within the ganglia, and when the immune system weakens, the virus reactivates and travels down sensory nerve axons, replicating in the skin areas innervated by these nerves, thus triggering herpes zoster [3]. Approximately 5% of herpes zoster patients develop postherpetic neuralgia [4, 5], which severely impacts the quality of life. When the VZV affects the ophthalmic branch of the trigeminal nerve, it can lead to herpes zoster ophthalmicus (HZO) [6]. In recent years, the incidence of HZO has been rising annually, with an increasing number of chronic and recurrent cases [7]. The recurrence rate over five years can reach as high as 25% [8]. Between 50% and 71% of HZO patients suffer from ocular complications, including keratitis, uveitis, and retinal necrosis [9]. Some patients experience delayed treatment due to late medical consultations, ultimately resulting in blindness. In this case, our hospital diagnosed a case of VZV-induced uveitis early using vitreous cavity puncture and metagenomic next-generation sequencing (mNGS) in an elderly male patient. Timely vitrectomy was performed, successfully preserving the patient’s vision. The detailed case report is as follows.

## Clinical data

### General information

**Fig. 1:**
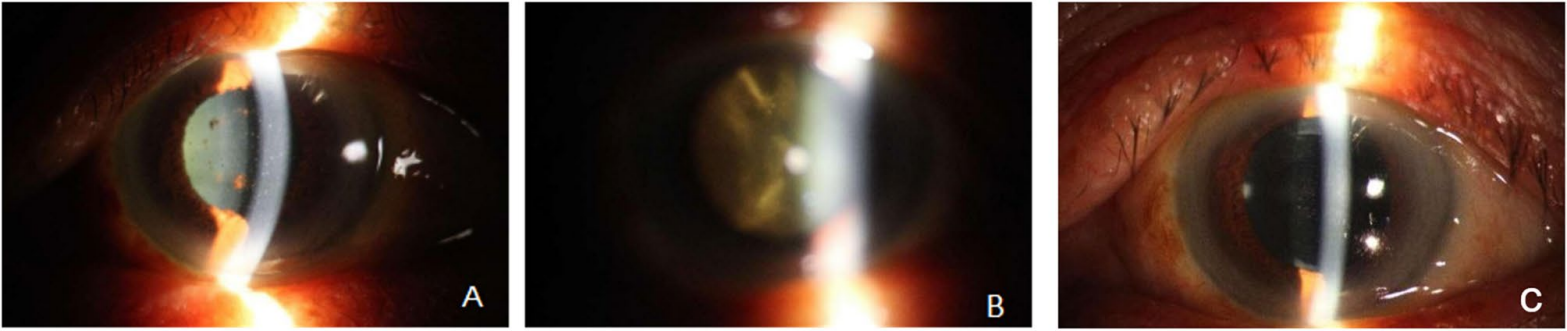
Anterior segment photography: posterior keratic precipitates (+++), floating cells in the anterior chamber (+++), partial posterior synechia of the iris (**A**); severe vitreous opacity with yellow-white floating debris (**B**); clear cornea, transparent anterior chamber, and in-position intraocular lens (**C**)

The patient is a 74-year-old male who presented to the neurology department with a 7-day history of dizziness and unsteadiness while walking. According to the findings of a neurologist’s physical examination, there is peripheral nerve damage.Other coordination tests did not reveal significant abnormalities. Upon further inquiry about his medical history, the patient reported a month-long history of decreased vision in the left eye. He also had a 17-year history of hypertension. An ophthalmology consultation was requested, and the ocular examination findings were as follows: right eye vision was 1.0, while the left eye showed hand movement perception at 10 cm. The right eye was normal, but the left eye exhibited slight conjunctival congestion, creamy keratic precipitates (KP) (+++), heavy cellular infiltration in the anterior chamber (+++) (According to SUN classification), partial posterior iris adhesions, lens opacity with visible pigment deposits on the iris, and severe vitreous opacities with yellowish-white floating debris.The optic disc structure can be vaguely seen in the fundus, but it is difficult to observe clearly.(Fig. 1A and B), with intraocular pressure of 11 mmHg in the right eye and 17 mmHg in the left eye. The patient denied a history of herpes zoster or any other significant medical history.

### Imaging and laboratory results

**Fig. 2:**
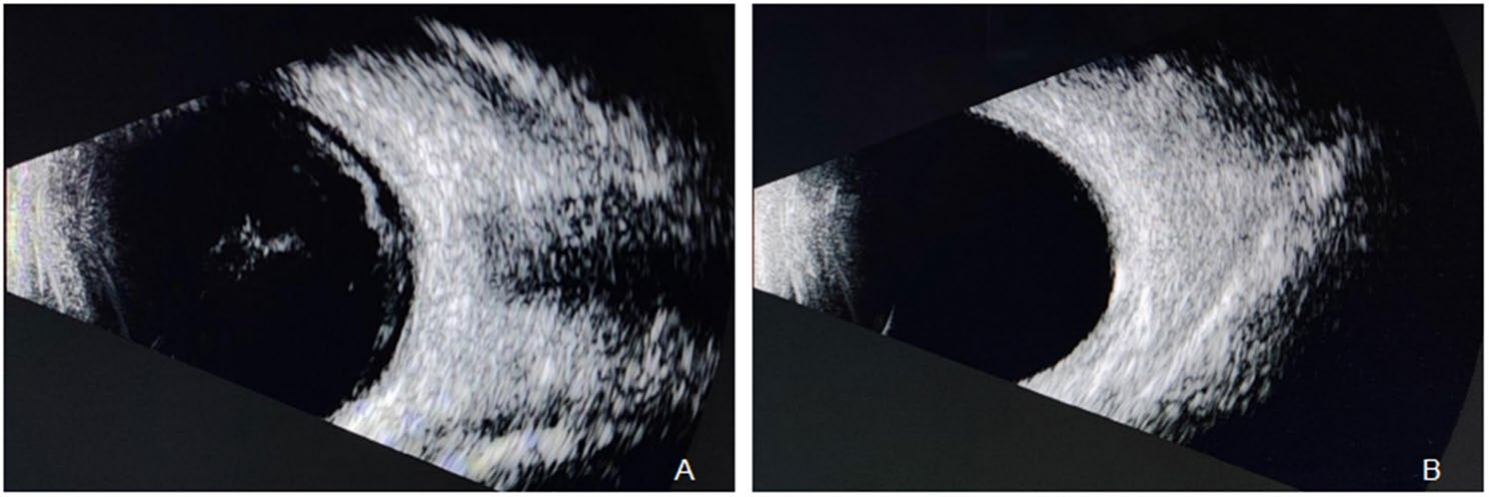
Left eye B-scan ultrasound: Severe vitreous opacity (**A**); Postoperative follow-up B-scan showing no significant vitreous opacity (**B**)

**Fig. 3:**
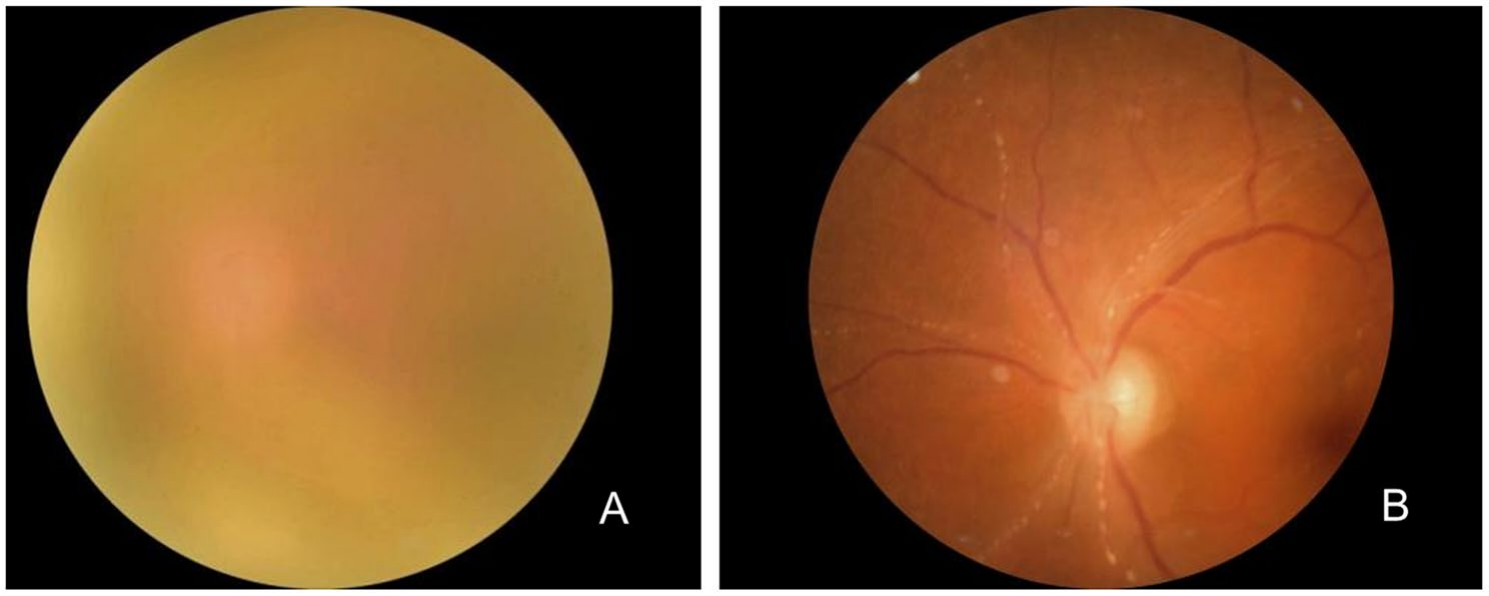
Fundus photography: The refractive interstitium in the left eye is unclear, and the optic disc structure is faintly visible (**A**); Fundus examination: After left eye surgery, the refractive interstitium is clear, and Kyrieleis plaques along the inner wall of the paraoptic artery can be seen. The macular structure is generally normal (**B**)

**Fig. 4:**
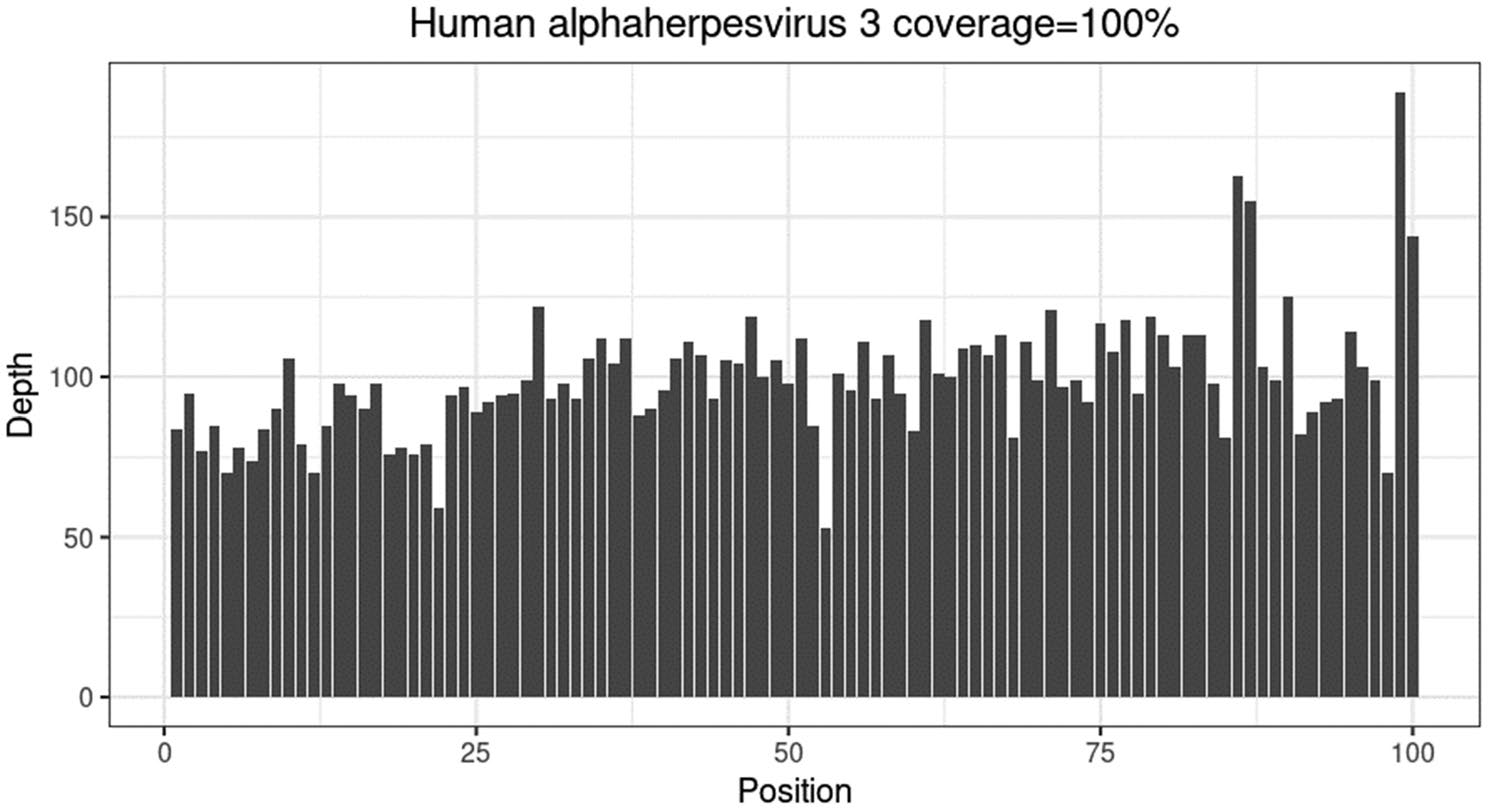
Metagenomic sequencing results of VZV: The x-axis represents the division of the microbial reference genome into 100 intervals, and the y-axis represents the sequence counts detected in each genomic interval

B-scan ultrasound of the left eye showed severe vitreous opacities (Fig. 2A). The optical coherence tomography (OCT) of the left eye revealed poor visualization of the retinal structures, with a faint view of the optic disc and an unclear fundus (Fig. 3A). mNGS of the vitreous fluid detected VZV (9939 sequence counts) and Epstein-Barr Virus (EBV) (40 sequence counts) (Fig. 4). After intravitreal injection of ganciclovir for antiviral treatment, cytokine testing of the vitreous fluid revealed significantly elevated levels of Interleukin-6 (IL-6), Interleukin-8 (IL-8), and Interleukin-10 (IL-10) (Table 1). The commonly used method for detecting interleukins in vitreous humor is flow cytometry, which can simultaneously detect multiple indicators (such as IL-6, IL-8, IL-10, etc.) with minimal sample size, high sensitivity, and strong specificity. It is commonly used to distinguish intraocular lymphoma from uveitis. Based on these results, the patient was diagnosed with VZV-induced uveitis.

### Systemic auxiliary examinations

Electromyography (EMG) of the left upper limb and both lower limbs revealed moderate peripheral nerve damage. Somatosensory evoked potentials (SSEP) of the lower limbs showed abnormalities in the peripheral segment. Vestibular function testing indicated positional vertigo attacks. Cerebrospinal fluid (CSF) biochemical analysis revealed: chloride 113 mmol/L (mildly decreased), CSF white blood cell count 90 × 10^6/L (↑), with 92% small lymphocytes (↑). The lumbar puncture revealed normal intracranial pressure. Based on the patient’s history of dizziness and unstable walking, as well as relevant examination results, it suggests viral encephalitis.

### Clinical diagnosis and treatment

Based on the patient’s medical history, ocular examination, and vitreous fluid metagenomic testing results, the diagnosis was confirmed as left eye uveitis. Since the patient initially presented to the neurology department, and the pathogen was not yet identified, the neurologists provided initial treatment focusing on neurotrophic therapy and improving microcirculation. After mNGS identified the pathogen, the treatment plan was promptly adjusted. The patient received intravenous administration of acyclovir (10 mg/kg every 8 h), local intravitreal ganciclovir injections (2 mg/0.1 ml) twice a week for two weeks, along with topical treatment using tobramycin-dexamethasone eye drops (4 times/day) and acyclovir eye drops (4 times/day). After two weeks, the anterior uveitis symptoms improved significantly, with inflammation in the anterior segment markedly reduced upon re-examination. However, visual acuity and vitreous opacity showed limited improvement, prompting the decision for cataract ultrasound phacoemulsification and vitrectomy. During surgery, large white floating debris was observed within the vitreous, fundus examination showed the refractive interstitium is clear, and Kyrieleis plaques along the inner wall of the paraoptic artery can be seen. The macular structure is generally normal (Fig. 3B). Based on intraoperative findings, a supplementary diagnosis of left retinal vasculitis was made. Postoperative treatment included intravitreal ganciclovir (2 mg/0.1 ml). On the first postoperative day, the patient’s left-eye visual acuity improved from hand movements at 10 cm to 0.2, with clear vitreous and a flattened retina. On postoperative day three, left-eye B-scan ultrasound showed no significant vitreous opacities (Fig. 2B), and anterior segment photography revealed a clear cornea, a transparent anterior chamber, and an in-position intraocular lens (Fig. 1C). The patient’s left-eye condition was stable and significantly improved. The patient was switched to oral valacyclovir (0.5 g twice daily) for two months.

**Table 1:** Vitreous fluid cytokine testing results

Test Item	Test Result	Unit	Reference Range
IL-6	620.82	pg/mL	≤ 5.4
IL-8	167.19	pg/mL	≤ 20.6
IL-10	48.63	pg/mL	≤ 12.9
IL-10/6	0.08		<1.0

## Discussion

VZV is the causative agent of both chickenpox and herpes zoster. It is commonly associated with peripheral nerve damage such as cranial nerve palsies, radiculitis, and central nervous system infections, including encephalitis, meningitis, vasculitis, and myelitis [10]. When VZV affects the ophthalmic branch of the trigeminal nerve, it can lead to HZO. Skin lesions in HZO primarily involve the side of the nose, nasal root, and nasal tip, a condition known as Hutchinson’s sign. The presence of Hutchinson’s sign increases the risk of ocular inflammation by 3 to 4 times [11]. Approximately 40%-60% of HZO patients develop anterior uveitis [12]. VZV-induced anterior uveitis typically presents acutely, progresses rapidly, and is often characterized by severe intraocular inflammation, with iris segmental atrophy and increased intraocular pressure commonly accompanying the condition [13]. However, there are few reported cases of VZV-induced uveitis and retinal vasculitis.

The etiology of most cases of uveitis remains unclear, with 50%-60% of patients having an unknown cause, and the condition is often recurrent. If not treated promptly, uveitis can cause irreversible damage to vision. Typically, bacterial and fungal cultures, as well as direct staining of intraocular fluid specimens, are considered the gold standards for pathogen detection [14, 15]. Traditional cultures require 3–5 days to yield results, with bacterial and fungal positivity rates of 22%-36% on direct microscopy and only 34%-60% on culture [16]. However, these traditional methods are unable to detect viral pathogens, making viral DNA detection a common alternative for identifying viral causes [17]. In recent years, several studies have demonstrated the significant advantages of mNGS in diagnosing infectious ocular diseases. For instance, Zhang et al. [18] reported a case of endogenous endophthalmitis caused by a fungus, where mNGS quickly identified Candida as the pathogen. Following targeted antifungal treatment, the patient’s vision significantly improved. Other studies have also confirmed that mNGS exhibits high sensitivity and specificity in detecting rare pathogens [19]. mNGS has a wide coverage and high detection rate, especially for rare and difficult to culture pathogens. Its limitation is that it cannot distinguish between infection and colonization contamination, and needs to be combined with clinical practice. It cannot be diagnosed solely based on results, has high costs, long cycles, no drug sensitivity information, and cannot directly guide medication.

In this case, the patient presented with generalized weakness in both lower limbs, with the main symptom being significant ocular inflammation. Considering the severity of eye inflammation, the decision was made to explore vitreous fluid for potential breakthroughs. Routine disinfection of the skin around the eyes, using a 1 ml syringe to inject 4 mm behind the temporal corneal edge, extracting 0.1 ml of vitreous humor, and sending it for testing of mNGS .The results from mNGS revealed the presence of VZV (9939 sequence counts) and EBV (40 sequence counts). Due to the higher incidence of EB virus infection in children/adolescents and the rarity of uveitis, which lacks absolute specific clinical manifestations, this patient has corneal lipoid KP (+++) and severe vitreous opacity with obvious Kyrieleis plaques. The symptoms are more consistent with Varicella Zoster Virus, and mNGS detection shows a lower number of EBV series. Therefore, this case is considered to be Varicella Zoster Virus. The patient is an elderly, single male, whose daily diet primarily consists of vegetarian food and pasta. He has long avoided consuming high-quality proteins such as milk, lean meats, and eggs, leading to a weakened immune system and an increased risk of infections.

Interleukin (especially IL-6, IL-8, IL-10) is a core indicator for determining the nature of infection. The levels of IL-6 and IL-8 in the intraocular fluid of patients with viral retinitis are significantly related to viral nucleic acid, indicating that the higher the viral load in the eye, the more severe the intraocular inflammatory response. The limitation of this case is that intraocular fluid cytokines were not detected before antiviral treatment. After two weeks of systemic and local antiviral treatment, the tested intraocular fluid cytokines showed significant increases in IL-6, IL-8, and IL-10 levels.

Based on the mNGS results, the treatment plan was formulated as follows: (1) Antiviral Therapy: Acyclovir, valacyclovir, and famciclovir are the main drugs used to treat HSV and VZV infections and can be applied topically or systemically [20]. In this case, the patient was given intravenous acyclovir (10 mg/kg every 8 h), local intravitreal ganciclovir injections (2 mg/0.1 ml) twice a week for two weeks, and topical eye drops. After discharge, the patient switched to oral valacyclovir for 3 months to consolidate the antiviral treatment. (2) Inflammation Suppression: Corticosteroids and nonsteroidal anti-inflammatory drugs (NSAIDs) can be used to control inflammation [21]. (3) Intraocular Pressure (IOP) Management: Simple IOP-lowering medications generally do not effectively control the pressure. Special attention is needed as prostaglandin analogs can activate herpesvirus, making them contraindicated [21]. (4) Symptomatic Treatment: Cycloplegics can be used to prevent posterior synechiae, while miotics should be avoided [22]. (5) Vitrectomy: For cases where conservative treatment is ineffective, vitrectomy can be performed to clear the lesion, break the blood-eye barrier, and improve drug penetration, thereby reducing the incidence of complications like retinal necrosis. Achieving optimal inflammation control before surgery is crucial for non-emergency treatments and surgical interventions for uveitis complications [23]. In this case, the patient’s visual acuity improved significantly postoperatively, with the vitreous cavity becoming clear, confirming the efficacy of vitrectomy in the treatment of infectious ocular diseases.

The case reported in this study utilized mNGS to quickly identify VZV, providing critical guidance for formulating the treatment plan. The use of intravenous acyclovir and local intravitreal injections ganciclovir, followed by timely vitrectomy when conservative treatment showed limited efficacy, effectively controlled the infection. This suggests that in patients with severe infectious ocular diseases, vitrectomy should be promptly considered after anti-infective therapy to achieve a favorable prognosis. The patient’s positive signs include anterior chamber planktonic cells and posterior iris adhesions, which are manifestations of uveitis. Kyrieleis plaques is a specific marker of viral vasculitis and is consistent with the symptoms of uveitis caused by VZV.After systemic and local antiviral treatment, as well as subsequent vitrectomy, the effect of this case was significant. Considering that the patient is an elderly and immunocompromised patient with a history of hypertension and no severe systemic infection symptoms, the use of hormones may cause blood pressure fluctuations, further leading to virus spread and worsening of infection. Therefore, the addition of adjuvant oral corticosteroids was not chosen for this case.

This case was conducted in accordance with the ethical principles of the 《Declaration of Helsinki》(as revised in 2013). It was approved by the Clinical Research Ethics Committee of the Hebei Chest Hospital, and written informed consent was obtained from the patient.

## Conclusion

VZV-associated uveitis is more likely to occur in immunocompromised individuals, and early diagnosis requires a combination of medical history and molecular diagnostic techniques. Metagenomic sequencing of intraocular fluid is an effective method for diagnosing uveitis caused by unidentified infectious pathogens. For patients with VZV-induced uveitis and severe vitreous opacities, vitrectomy combined with systemic antiviral therapy can effectively control the infection. The successful treatment of this case highlights the importance of multidisciplinary collaboration and reminds us that early application of intraocular fluid metagenomic sequencing is essential when encountering uveitis of unknown infectious etiology, enabling timely and targeted treatment.

## Data Availability

All data generated or analyzed during this study are included in this published article and its supplementary information files.
